# Individual word activation and word frequency effects during the processing of opaque idiomatic expressions

**DOI:** 10.1177/17470218211047995

**Published:** 2021-10-04

**Authors:** Ferdy Hubers, Catia Cucchiarini, Helmer Strik, Ton Dijkstra

**Affiliations:** 1 Centre for Language Studies, Radboud University, Nijmegen, The Netherlands; 2 Centre for Language and Speech Technology, Radboud University, Nijmegen, The Netherlands; 3 Donders Institute for Brain, Cognition and Behaviour, Radboud University, Nijmegen, The Netherlands

**Keywords:** Idioms, priming, word naming, word frequency, form-meaning interaction

## Abstract

Idiom processing studies have paid considerable attention to the relationship between idiomatic expressions as a whole and their constituent words. Although most research focused on the semantic properties of the constituent words, their orthographic form could also play a role in processing. To test this, we assessed both form and meaning activation of individual words during the processing of opaque idioms. In two primed word naming experiments, Dutch native speakers silently read sentences word by word and then named the last word of the sentence. This target word was embedded in either an idiomatic or a literal context and was expected and correct in this context (COR), semantically related (REL) to the expected word, or unrelated (UNREL) to the expected word. The correct target word in the idiomatic context was always part of an opaque idiom. Faster naming latencies for the idiom-final noun than for the unrelated target in the idiomatic context indicated that the idiom was activated as a whole during processing. In addition, semantic facilitation was observed in the literal context (COR < REL < UNREL), but not in the idiomatic context (COR < REL = UNREL). This is evidence that the idiom-final noun was not activated at the meaning level of representation. However, an inhibitory effect of orthographic word frequency of the idiom-final noun indicated that the idiom-final noun was activated at the form level. These results provide evidence in favour of a hybrid model of idiom processing in which the individual words and the idiom as a whole interact on form and meaning levels of representation.

## Introduction

There is a long-standing tradition of research on idiom processing in psycholinguistics. An important question in this domain is whether idiomatic expressions, such as “kick the bucket” and “spill the beans” are stored in the mental lexicon as a whole or not. A considerable body of evidence has demonstrated that, to some extent, this indeed is the case (e.g., Bobrow & Bell, 1973; Cutting & Bock, 1997; Rommers et al., 2013; Sprenger et al., 2006; Swinney & Cutler, 1979; van Ginkel & Dijkstra, 2020).

However, even if an idiomatic expression is stored as a whole, it is still composed of parts: namely, its individual words. This leads to the question of how processing is affected by the relation between those parts (words) and the idiomatic expression as a whole. Take, for instance, an idiomatic expression that can also be literally interpreted: “to kick the bucket.” In its literal interpretation, the meaning of the target word “bucket” must be integrated in the literal meaning of the phrase as a whole. How quickly and smoothly this can be done will co-depend on lexical properties of the word “bucket”, for instance, its word frequency. When the word has a higher frequency, lexical-semantic integration will likely take place more quickly. However, when one must understand the idiom “to kick the bucket” in its sense “to die” the meaning of the individual word “bucket” is actually irrelevant and may interfere with that of the idiom as a whole. Nevertheless, to verify that the idiom “to kick the bucket” is indeed being presented, the word form of “bucket” must still be identified. As a consequence, during the interpretation of the expression “to kick the bucket” as an idiom, it would be best to process the word “bucket” at the form level, but to avoid activation of its meaning—if that is at all possible.

Previous studies investigating the role of the individual words during idiom processing have mainly focused on the activation of their semantics (e.g., Cutting & Bock, 1997; Rommers et al., 2013; Sprenger et al., 2006). However, it seems likely that the processing difficulty of the idiom as a whole co-depends on the properties of the target word related to its form, such as its frequency relative to the frequency of the idiomatic expression as a whole. In the idiomatic case, a higher target word frequency should actually lead to slower processing of the idiom, reflecting competition between the idiom as a whole and the target word at the form level of representation. However, the role of individual word frequency during idiom processing has received only little research attention (Cronk et al., 1993; Libben & Titone, 2008; van Ginkel & Dijkstra, 2020).

This study aims to fill this gap by investigating how the individual words and the idiom as a whole interact both orthographically and semantically during idiom processing.

To set the stage for the presentation of our study, we first review previous studies on the activation of individual words during idiom processing. First, we focus on research that addressed individual word activation at the semantic level and the way this is affected by properties related to the semantics of the idiom as a whole. Next, we review the limited number of studies that have examined the activation of individual words during idiom processing at the form level by considering word frequency effects.

### Semantic effects of individual words during idiom processing

Early studies argued that individual word meanings are not activated during idiom processing and that idioms are stored as a whole in the mental lexicon (Bobrow & Bell, 1973; Gibbs, 1980; Swinney & Cutler, 1979). These findings formed the basis for non-compositional models of idiom processing. According to the Idiom List Hypothesis by Bobrow and Bell (1973), idiom comprehension requires a special idiom mode of processing. Once participants are in this idiom processing mode, the individual word meanings do not affect processing. The Direct Access Hypothesis, proposed by Gibbs (1980), does not identify different processing modes, but suggests that an idiom’s figurative meaning can be directly accessed in the mental lexicon without an analysis of the literal meaning. Only if idiomatic processing fails will phrases be analysed literally.

However, later studies have shown that the semantics of the individual words in idiomatic expressions do contribute to their figurative interpretation. This led to the development of compositional models of idiom processing (Cacciari & Glucksberg, 1991; Gibbs, Nayak, Bolton, & Keppel, 1989; Nunberg, 1979). Here, individual words are activated during idiom processing and an idiom’s figurative meaning is retrieved by combining the semantics of the individual words. A prominent compositional model is the Idiom Decomposition Hypothesis (Gibbs, Nayak, & Cutting, 1989).

More recent studies suggest that literal and figurative processing run in parallel, and depending on the time-course and properties of the idiom, the meanings of the individual words may be activated or not (Beck & Weber, 2016; Cacciari & Tabossi, 1988; Cutting & Bock, 1997; Libben & Titone, 2008; Sprenger et al., 2006; Titone et al., 2015; Titone & Libben, 2014). The Configuration Hypothesis by Cacciari and Tabossi (1988) is such a hybrid model, in which idiom-containing sentences are initially processed compositionally. After the idiom is identified (the idiom recognition point), the individual word meanings are suppressed and the figurative meaning becomes available. Sprenger et al. (2006) proposed a hybrid model of idiom production in which idiomatic expressions have separate representations (*superlemmas*) that are connected to simple word lemmas, on the one hand, and to idiomatic meaning representations, on the other hand. The superlemmas can be accessed by activating the simple lemmas of the component words. The extent to which the individual word meanings are activated may be modulated by properties related to the semantics of the idiom as a whole. This idea has been put forward by Libben and Titone (2008) in their Constraint-Based Model. Idiom properties such as familiarity and predictability, which are related to direct retrieval, may affect early stages of idiom comprehension, whereas decomposability or transparency may become important later on (Titone et al., 2015; Titone & Libben, 2014).

Many of the early studies that found support for purely compositional or non-compositional models of idiom processing did not directly consider the activation of individual word meanings; instead, they focused on the processing of idiomatic and literal phrases as a whole using phrase classification tasks (e.g., Gibbs, Nayak, & Cutting, 1989; Swinney & Cutler, 1979). Later studies did examine the semantic activation of individual words during idiom processing using priming paradigms by assessing semantic spreading activation (Cacciari & Tabossi, 1988; Rommers et al., 2013; van Ginkel & Dijkstra, 2020). If the meaning of a single word (that is part of the idiom) is activated, it should co-activate words that are semantically related to this word. Finding a facilitatory effect for words that are semantically related to the individual component words (the literal meaning) implies that those component words are semantically activated themselves. In contrast, the absence of this spreading activation effect during idiom processing has been taken as evidence for the suppression of the individual words.

A study based on this argumentation is the combined response time (RT) and electroencephalography (EEG) study by Rommers et al. (2013). They investigated the activation of literal word meanings during the processing of Dutch opaque idioms. Rommers et al. (2013) specifically considered opaque idioms, because in this type of idiom the individual word meanings are not related to the idiom’s figurative meaning. Participants were presented with idiomatic and literal sentence contexts in a rapid serial visual representation (RSVP) fashion. The idiomatic sentence contexts always included an idiom (e.g., “*tegen de lamp lopen*” lit. “walk against the lamp” which means “to get caught”). Following Federmeier and Kutas (1999), the critical word was a correct and expected word (lamp), a word that was semantically related to the expected word (candle), or a word that was semantically unrelated to the expected word (fish). In the idiomatic sentence contexts, the correct and expected word was always a noun that was part of the idiom. The same critical words were used in literal sentence contexts in which the correct and expected word was equally predictable (e.g., “After lunch the electrician screwed the new light bulb into the lamp yesterday”). In the behavioural version of the experiment, participants performed a lexical decision task on the critical words, while in the EEG version of the experiment, no task was involved and N400 effects were measured. In the literal sentence contexts, a graded pattern was observed in terms of RT and N400 effects: The fastest response was to the correct and expected word (COR) and it elicited the smallest N400 effect, followed by the semantically related (REL) and unrelated (UNREL) word, respectively. In the idiomatic sentence context, however, no difference was observed between the REL and UNREL conditions. Apparently, in the idiomatic sentence context, spreading activation from the expected to the semantically related word was absent. Rommers et al. (2013) concluded that “when reading predictable and opaque idiomatic expressions, for which literal word meanings are irrelevant, the processing of literal word meanings can to some extent be ‘switched off’” (p. 775).

### Orthographic effects of the individual words during idiom processing

If individual words are not accessed during idiom processing, effects of orthographic properties of these words, such as word frequency, should be absent too. If the individual word forms are activated, but activation is not strong enough to access their semantics and subsequently co-activate semantically related words, orthographic effects, such as word frequency, might nevertheless be visible. However, the activation of the orthography of individual words in idiom processing has received limited attention. Only three studies have examined this issue by investigating the role of individual word frequency in idiom processing (Cronk et al., 1993; Libben & Titone, 2008; van Ginkel & Dijkstra, 2020).

Cronk et al. (1993) investigated the role of word frequency in relation to idiom familiarity in a self-paced reading paradigm. Idiom familiarity was obtained through a norming study, in which participants rated how often the phrase (the idiom) is heard used figuratively on a 5-point scale. Frequencies of the idioms’ component words were taken from Kučera and Francis (1967) and were averaged per idiom. Cronk et al. (1993) found that high-familiar idioms were read more quickly than low-familiar idioms, and that this effect was modulated by word frequency: The familiarity effect was larger for idioms containing high-frequency words as opposed to idioms containing low-frequency words. More specifically, mean reading times per word were much faster for high-familiar idioms consisting of high-frequency words than for high-familiar idioms containing low-frequency words and low-familiar idioms. These findings suggest that the individual words do contribute to the figurative meaning. If the idiom component words are highly frequent, the figurative meaning may be retrieved faster than in the case of component words of low frequency.

In a series of three experiments, Libben and Titone (2008) investigated the role of various idiom properties, such as familiarity, decomposability, and literality, on idiom processing and the effects of verb and noun frequency in idioms with a “she [verb] × [noun]” structure. No effect of noun frequency was found on the RTs for idioms. Verb frequency, however, turned out to negatively affect idiom processing, indicating that, paradoxically, idioms with low-frequency verbs were recognised more quickly than idioms with high-frequency verbs. Based on their results, Libben and Titone (2008) argued that infrequent verbs are probably more predictive of idiomatic completions than high-frequency verbs and therefore lead to a processing advantage.

In a priming study, van Ginkel and Dijkstra (2020) presented participants with idiomatic expressions as primes after which target words followed that were figuratively related to the idiom as a whole (FIG condition), semantically related to the literal word at the end of the idiom (LIT condition), or unrelated to the idiom and the idiom-final noun (UNREL condition). Participants were instructed to perform a lexical decision on the target words. Van Ginkel and Dijkstra (2020) found an idiom priming effect in the FIG condition compared with the UNREL condition, which they interpreted as evidence in support of the hypothesis that the representations of idioms are activated as a whole. However, they also found that literal word meanings were activated, as reflected by a priming effect for the LIT condition compared with the UNREL condition. Interestingly, in the LIT condition, a word frequency effect of the idiom-final noun was observed that was absent in the FIG and UNREL conditions. More specifically, idiom-final noun frequency negatively affected reaction times on target words semantically related to the idiom-final noun: Higher frequencies resulted in slower reaction times. Van Ginkel and Dijkstra (2020) suggested that this inhibition effect might be due to conflicting processes. On the one hand, the idiomatic reading leads to strong activation of the idiom representation as a whole, while, on the other hand, the literal words also become activated. If the idiom-final word is of high frequency, it is more difficult to suppress its activation than when it is of low frequency. Thus, literal words are not fully suppressed.

Although the studies reviewed above found that individual words are activated during idiom processing, at least at the orthographic level of representation, they showed mixed results with respect to the role of individual word frequency. Cronk et al. (1993) reported a facilitatory effect of word frequency. In contrast, Libben and Titone (2008) found an inhibitory effect of verb frequency on idiom processing, but no effect of idiom-final noun frequency, while van Ginkel and Dijkstra (2020) observed an inhibitory effect of idiom-final noun frequency on idiom processing. These inconsistent results may be due to the different tasks used in these studies. In line with this observation, van Ginkel and Dijkstra (2020) proposed a context-sensitive hybrid task-dependent processing account, in which literal and figurative processing run in parallel. In this account, the crucial element is the moment at which the target word is presented in relation to the sentence as a whole.

### The present study

To gain more insight into the mixed results of earlier studies, we investigated the role of the individual words during idiom processing at both the semantic and orthographic level of representation. With respect to our design, we were inspired by the study of Rommers et al. (2013), who used an RSVP paradigm to investigate the activation of the idiom-final nouns of opaque idiomatic expressions in highly biasing contexts. They found that the activation of the idiom-final nouns was suppressed in terms of their semantics. However, the individual idiom-final words are expected to be activated to some extent because the word form needs to be identified to complete the idiom. Although Rommers et al. (2013) observed no activation of the semantics of the individual idiom-final words during the processing of opaque idiomatic expressions in highly biasing context sentences, effects of lexical properties of the idiom-final nouns related to the orthography, such as word frequency, might still be present.

To investigate this issue, we used the same paradigm as Rommers et al. (2013). We adopted this particular design with longer presentation times because this way it would also be suitable for a potential EEG study to accompany it and because it has been applied in other RT studies as well (e.g., Rommers et al., 2013; van Ginkel & Dijkstra, 2020). However, instead of a lexical decision task, which also taps into semantic information, we used a word naming task, which relies more on word form (orthography and phonology). By focusing more on the word form, effects of the individual word semantics are expected to be reduced, whereas word frequency information, associated with the word form, may become available anyway.

Participants were presented with target words embedded in an idiomatic context sentence or a literal context sentence (see Table 1). These target words were the correct and expected target words given the context (COR condition), semantically related to the expected target word (REL condition), or semantically unrelated to the expected target word (UNREL condition). The expected target word (COR) in the idiomatic context was always a noun that was part of an idiom, while the literal context sentences contained a bias to the literal meaning of this same target word.

**Table 1.** Dutch example sentences of experimental items with their English translations.

Condition	Example sentence
*Idiomatic*	
COR	De getrainde dief liep uiteindelijk toch tegen de lamp
	*The trained thief eventually walked against the lamp*
REL	De getrainde dief liep uiteindelijk toch tegen de warmte
	*The trained thief eventually walked against the warmth*
UNREL	De getrainde dief liep uiteindelijk toch tegen de helm
	*The trained thief eventually walked against the helmet*
*Literal*	
COR	Het kind kan niet slapen zonder licht van een kleine lamp
	*The child cannot sleep without light of a little lamp*
REL	Het kind kan niet slapen zonder licht van een kleine warmte
	*The child cannot sleep without light of a little warmth*
UNREL	Het kind kan niet slapen zonder licht van een kleine helm
	*The child cannot sleep without light of a little helmet*

Target words are underlined. The figurative meaning of the idiomatic context with the correct target word (lamp) is “The trained thief eventually got caught.”

We made three predictions. First, we hypothesised that the idiom as a whole has its own separate representation in the mental lexicon that is activated and recognised during processing. If this is the case, we should observe faster responses to the correct and expected target word (COR) in the idiomatic context (idiom-final noun), a target word semantically unrelated to the literal meaning of the expected item (UNREL), and a target word semantically related to the literal meaning of the expected item (REL). These results would also be in line with other studies using the RSVP paradigm in combination with highly biasing context sentences (Federmeier, 2007; Federmeier et al., 2002; Federmeier & Kutas, 1999; Rommers et al., 2013).

Second, because the idiom-final word’s form characteristics must be retrieved to integrate it successfully into the idiomatic context, we expected to observe activation of the idiom-final noun at the orthographic form level in terms of word frequency. More specifically, we expected the idiom’s component words to compete with the idiom as a whole at the form level. As a consequence, slower naming latencies in the idiomatic context should arise for conditions with higher individual target word frequencies, in line with van Ginkel and Dijkstra (2020).

Third, because we used opaque idiomatic expressions in which the individual word meanings do not contribute to the figurative meaning, and presented them in a highly idiomatically biasing context, we expected only limited activation of the semantics of the correct target word in the idiomatic context. Thus, words that are semantically related to the literal interpretation of idiom-final noun would not be activated either. In line with Rommers et al. (2013), we therefore predicted that in the idiomatic context, the naming latencies to the semantically related target word and the unrelated target would not differ. However, in the literal context, the semantically related target word would be responded to faster than the unrelated target word because there the correct target word would be fully activated, spreading activation to semantically related words.

## Experiment 1

### Methods

#### Participants

Thirty-two native speakers of Dutch participated in the first experiment (24 females and 8 males). They were between 19 and 33 years old (*M* = 23.7; *SD* = 3.63) and had a normal or corrected-to-normal vision. They received compensation for participation in terms of a gift card or participant credits. Participants provided a written informed consent before the start of the experiment. This study was ethically assessed and approved by the Ethics Assessment Committee (EAC) of the Faculty of Arts of Radboud University Nijmegen (number 3382).

#### Materials and design

##### Idiom selection

We compiled a database of 374 Dutch idiomatic expressions that were rated by 390 native speakers of Dutch on different dimensions, such as Transparency, Familiarity, and Imageability. The ratings were found to be highly reliable (Hubers et al., 2018, 2019). We selected 30 opaque idiomatic expressions from this database as a basis for the experimental sentences. The idiomatic expressions included in this study had a mean transparency rating of 2.22 on a scale from 1 to 5 (*SD* = 0.35; range = 1.31–2.61) and were said to be encountered quite frequently in daily life (*M* = 3.00; *SD* = 0.75; range = 2.04–4.76; scale, 1–5).

##### Sentence construction

The materials consisted of 180 experimental sentences (30 sets of 6 sentences) and 60 filler sentences. The target word was always the last word of the sentence. In the filler sentences, the target word was a noun in a literal context. In the experimental sentences, however, the target word was either a noun that was part of an idiom (idiomatic context) or the same noun embedded in a literal context. The experiment involved a within-subject design with the variables Context (Idiomatic and Literal) and Condition (COR, REL, and UNREL).

Because of the within-subject design, we created three different sentences based on each of the 30 idioms in each context (Idiomatic and Literal). Subsequently, three versions of the same sentence were created by changing the target word. The target word was the expected and correct word given the context (COR), a word that was semantically related to the expected word (REL), or a word that was semantically unrelated to the expected word (UNREL). See Table 1 for example stimuli.

The materials were divided into three master lists containing 210 sentences: 180 experimental sentences (90 idiomatically biasing sentences and 90 literally biasing sentences with COR, REL and UNREL evenly distributed; 30 sentences of each condition) and 30 filler sentences with an expected target word only. Each participant received a pseudo-randomisation of one of the three lists.

##### Target word selection

The semantically related target words were obtained from the word association database from De Deyne and Storms (2008) when possible. If no appropriate word associations were available, we thought of semantically related words ourselves. In a pre-test, all potential REL and UNREL target words were tested with respect to their semantic relatedness to the expected target word (COR). The pre-test consisted of a rating task in which participants had to indicate to what extent word pairs were related on a 5-point Likert-type scale (ranging from 1 “not related at all” to 5 “highly related”). In total, 79 Dutch native speakers participated in two versions of the pre-test. We selected REL words if the average association score was above 3.5 and UNREL words if the association score was below 2.5. The REL words included in the experiment had an average association score of 4.33 (*SD* = 0.37; range = 3.60–4.93). The average association score for the UNREL words included in the experiment was 1.49 (*SD* = 0.35; range = 1.04–2.14).

Target word frequency and target word length in letters were matched across conditions. We extracted the word frequencies per million from SUBTLEX-NL (Keuleers et al., 2010). The conditions (COR, REL, and UNREL) did not significantly differ in terms of log-transformed word frequency, *F*(2, 87) = 0.055, *p* = .947 (*M* = 2.75; *SD* = 0.64). The conditions did not significantly differ in terms of target word length, *F*(2, 87) = 0.920, *p* = .083 (*M* = 4.86; *SD* = 1.30).

We controlled for the initial sound of the target words, given that in word naming especially fricatives and plosives may trigger the voice key later than, for example, nasals, even if the articulatory onset of these phonemes takes place at the same time (e.g., Duyck et al., 2008; Tyler et al., 2005). In line with Duyck et al. (2008), we divided the target words into five categories depending on their initial phoneme: vowels, fricatives, nasals, plosives, and approximants. The target words were selected in such a way that within each condition (COR, REL, and UNREL), the phonetic categories of the initial sounds were similarly distributed, especially with respect to fricatives and plosives.

##### Cloze probability

We controlled for the cloze probability of the expected target words (COR) in both the idiomatic and literal contexts. To this end, we conducted a pre-test including 219 potential experimental sentences without the final word (the target word). These sentences were divided over two lists. Participants were asked to fill in the first word that came to mind upon reading the sentences. In total, a group of 17 participants carried out this first version of the cloze test (age *M* = 20.6; *SD* = 1.6; female = 14). A subset of the sentences was adapted and tested again. The second version of the cloze test contained both the adjusted sentences and the sentences that had been already tested. The design and procedure of this test were the same as before. In total, 38 people participated (31 females). They were on average 32.6 years old (*SD* = 12.7). In a third version of the cloze test, the remaining set of 43 adapted sentences were tested by a group of 20 participants (age *M* = 31.3; *SD* = 12.7). The experimental sentences in both the literal and the idiomatic contexts had comparable cloze probabilities (LIT: M = 0.82, *SD* = 0.15; IDIOM: *M* = 0.83; *SD* = 0.16), *t*(178) = 0.0387, *p* = .699.

##### Sentence plausibility

To obtain information about the plausibility of the sentences containing a violation (REL and UNREL), we carried out a sentence plausibility test. An independent group of 32 native speakers of Dutch were asked to assess whether the sentences were plausible on a scale ranging from 1 (“not plausible at all”) to 7 (“highly plausible”). All materials were divided over three lists containing 180 sentences (90 literally biasing sentences and 90 idiomatically biasing sentences with COR, REL, and UNREL evenly distributed). The participants were randomly assigned to the list, resulting in almost evenly distributed groups of participants per list (cf. 9, 11, and 12 participants). Half of the participants in each group received the list in reverse order. Table 2 provides the mean plausibility ratings for the experimental sentences. The literal contexts were rated as more plausible than idiomatic contexts, *F*(1, 31) = 126.82; *p* < .01. In addition, Condition, *F*(1.54, 47.60) = 1048.04; *p* < .01, and the interaction effect of Context and Condition, *F*(1.67, 51.82) = 48.63; *p* < .01, were significant. Simple effect analyses showed that COR, REL, and UNREL significantly differed from each other in both the Literal and Idiomatic contexts.

**Table 2.** Mean plausibility ratings and *SD*s for the experimental sentences (scale 1–7).

Condition	Context	
	Literal	Idiomatic
COR	6.5 (0.5)	5.9 (0.9)
REL	3.6 (0.6)	1.9 (0.6)
UNREL	1.6 (0.3)	1.4 (0.4)

See Supplementary Materials for the idiomatic expressions included in the experiment and their corresponding target words.

#### Procedure

The participants were tested in a soundproof booth. The experiment was programmed in PsychoPy (Peirce, 2007). Word naming was recorded with a head-mounted microphone (SHURE WH-20-XLR), and naming latencies were calculated by the PsychoPy voice-key module (Peirce, 2007). Because of potential problems with PsychoPy online voice-key measurements, we used MATLAB (MathWorks, 2016) to check the exact speech onset times afterwards based on the target word recordings.

The experiment consisted of two parts: (1) the familiarisation phase and (2) the main experiment. For the first part, participants were told to read idiom–meaning pairs. Although we selected idioms for our experiment that were relatively frequent, we included a familiarisation phase prior to the main experiment because we intended to conduct this experiment also with L2 learners of Dutch, who are generally less familiar with the idioms. For this group, we wanted to increase the likelihood that participants recognised the idioms as such. As for the main experiment, participants were instructed that they would read sentences presented word by word on the screen, with the last word of each sentence presented in red. They were asked to read aloud the red word as quickly as possible. Furthermore, participants were instructed that every now and then they would be presented with comprehension questions about the sentence directly preceding the question. They were asked to answer the question with yes or no by pressing the corresponding buttons on the button box. In this way, we forced the participants to actually read the sentence context preceding the target word.

In the familiarisation phase, all 30 idiomatic expressions included in the main experiment were presented to the participants along with their meanings. The idiomatic expressions were presented at the centre of the screen in white on a black background with the meaning of the idioms directly below them. After 30 s, the next idiom–meaning pair automatically appeared on the screen. No explicit task was formulated. This part of the experiment took approximately 5 min.

The main experiment started with a practice phase consisting of 11 practice trials and 3 comprehension questions for the participants to get used to the task. After the practice phase, they had the opportunity to ask questions if anything was unclear.

A trial started with a fixation cross that was presented for 500 ms, followed by a blank screen of 300 ms. Subsequently, a sentence was presented in a word-by-word fashion. The words were presented at the centre of the screen in white on a black background. Each word was displayed for 300 ms, after which a blank screen was presented for 300 ms. The last word of the sentence, the target word, was presented in red and disappeared after 2,500 ms or when the voice-key triggered. The next trial was presented automatically 2,500 ms after the onset of the target word.

After the main experiment, participants filled in a background questionnaire and were tested on their knowledge of the idiomatic expressions included in the experiment by means of an open-ended question about the idiom meanings. In total, it took participants 1 hr to complete the experiment.

#### Data analysis

We performed linear mixed-effects regression analyses to analyse the naming latencies. These analyses were conducted in the statistical software package “R” version 3.4.0 (R Development Core Team, 2008), and the R packages “lme4” (Bates et al., 2015), “lmerTest” (Kuznetsova et al., 2017) and “effects” (Fox, 2003) were used. The models were built in a forward manner, starting off with a basic model including a random intercept for participants and the variables of interest (Context and Condition). Subsequently, we added different predictors to the model (random and fixed factors) one by one based on theory. After adding a predictor, we examined whether the model fit improved. If this was not the case, we decided not to include this predictor in the model. The final model is reported in this article.

### Results

Naming errors and trials with naming latencies shorter than 360 ms were removed from the data (2.8 %). Three participants were removed because of poor performance on the comprehension questions (<70% correct). Responses at 2.5 *SD*s from the mean were removed on the participant and item level (2%). The average naming latencies and *SD*s per Context and Condition are presented in Table 3.

**Table 3.** Mean naming latencies and *SD*s in Experiment 1.

Condition	Context	
	Literal	Idiomatic
COR	579 (117)	566 (124)
REL	607 (119)	592 (117)
UNREL	614 (121)	591 (112)

We performed a linear mixed- effects regression analysis to analyse the data. The log-transformed reaction times were used as the dependent variable. In our final regression model, we included the following predictors as fixed effects: (1) Context (Idiomatic and Literal), (2) Condition (COR, REL, and UNREL), (3) Trial Number, (4) Cloze Probability, (5) Sentence Plausibility, (6) Initial Sound (Vowels, Plosives, Fricatives, Approximants, and Nasals), (7) Target Word Frequency (Logged), (8) Idiom Transparency, (9) Context × Condition, and (10) Context × Idiom Transparency.

We included Initial sound as a covariate in our analysis because voice keys are known to be less sensitive to words starting with plosives and fricatives compared with words starting with other sounds (Duyck et al., 2008; Tyler et al., 2005). By including this factor, we are able to account for variation in the data that otherwise would be incorporated in the effects of our predictors of interest.

In addition, we included target word (intercept only) and participants (intercept and random slope of Trial Number) as random effects. We included target word as an item-related random effect instead of idiom because the target words occurred in both the literal and the idiomatic contexts, while the idioms were only presented as such in the idiomatic context. To be able to better interpret the results of the regression model in the light of our hypotheses, we changed the reference categories for the categorical predictors to Literal (for Context), Fricatives (for Initial Sound), and REL (for Condition). The variable Trial Number was standardised, and Idiom Transparency was mean centred. The variable Cloze Probability reflected the cloze probability of the correct target word and was used as a measure of predictability. The model is presented in Table 4.

**Table 4.** Regression model Experiment 1 with logged naming latencies as the dependent variable.

Fixed effects		Beta	*SE*	*t* value	
(Intercept)		6.44500	0.02960	217.905	***
Trial Number		0.00420	0.0043	0.980	
Cloze Probability		−0.09240	0.0275	−5.851	***
Sentence Plausibility		−0.00470	0.0023	−2.047	*
Initial Sound (Vowels)		0.06710	0.0144	4.453	***
Initial Sound (Plosives)		0.05120	0.0087	5.641	***
Initial Sound (Nasals)		0.01240	0.0134	0.883	
Initial Sound (Approximants)		0.06040	0.0146	3.955	***
Target Word Frequency		−0.00630	0.0027	−2.205	*
Context (Idiomatic)		−0.03220	0.0079	−4.061	***
Condition (COR)		−0.02700	0.0249	−2.170	*
Condition (UNREL)		0.00790	0.011	0.703	
Idiom Transparency		0.00260	0.014	0.187	
Context (Idiomatic) × Condition (COR)		−0.00170	0.0101	−0.170	
Context (Idiomatic) × Condition (UNREL)		−0.00500	0.0103	−0.483	
Context (Idiomatic) × Idiom Transparency		−0.04180	0.0122	−3.478	***
Random effects		Variance	*SD*	Corr.	
Target word	Intercept	0.00086	0.0294		
Participant	Intercept	0.01580	0.1257		
	Trial number	0.00042	0.0203	0.24	
Residual		0.01968	0.1403		

* *p* < .05. **p < .01. ***p < .001.

The analyses revealed no significant interaction effect between Context and Condition. The differences between COR and REL (β = −0.002, *SE* = 0.030, *p* > .05) and REL and UNREL (β = −0.005, *SE* = 0.010, *p* > .05) in the Idiomatic and Literal contexts were similar. Naming latencies in response to the correct target word were significantly faster than to the related target words in the literal context (β = −0.027, *SE* = 0.030, *p* < .05). Surprisingly, the naming latencies for the semantically related target words did not significantly differ from those of the unrelated target words in the literal context (β = 0.008, *SE* = 0.010, *p* = .483).

Similar results were found for the effect of Condition in the idiomatic context. A relevelled version of the model showed significantly faster responses to the correct target words in the idiomatic context than to the semantically related target words (β = −0.029, *SE* = 0.014, *p* < .05) and no significant differences between the semantically related and unrelated target words (β = 0.003, *SE* = 0.010, *p* > .05). A general facilitatory effect of Target Word Frequency was found (β = −0.006, *SE* = 0.003, *p* < .05): higher target word frequencies were associated with faster naming latencies. Moreover, facilitatory effects of Cloze Probability (β = −0.092, *SE* = 0.016, *p* < .001) and Sentence Plausibility (β = −0.005, *SE* = 0.002, *p* < .05) were found. Sentences in which the correct target word was more predictable elicited faster naming latencies than sentences in which the correct target word was less predictable. This effect was not modulated by Context and Condition.

Idiom Transparency turned out to affect naming latencies in the idiomatic context only as indicated by the significant interaction effect between Context and Idiom Transparency (β = −0.042, *SE* = 0.012, *p* < .001). A relevelled version of the model showed a facilitatory effect of Idiom Transparency in the idiomatic context irrespective of the Condition (β = −0.052, *SE* = 0.014, *p* < .001): the more transparent an idiom, the faster the naming latencies in response to the target word. Adding other idiom properties, such as idiom imageability, did not significantly improve the model fit. Therefore, these properties were not included in the regression model.

### Discussion

For the idiomatic context, we found faster naming latencies for the correct target word than for the semantically unrelated target words. This is in line with our hypothesis that idiomatic expressions as a whole are activated and recognised, and suggests that idioms have a separate representation in the mental lexicon. In the literal context, correct target words were also named faster than semantically unrelated target words, which indicates that lexical-semantic integration takes place more quickly for these target words because of the literally biasing context. Both results are in line with the findings of Rommers et al. (2013).

In line with our second prediction, target words seemed to be activated at the form level of representation, as observed by a facilitatory effect of orthographic target word frequency. This fits in with the general finding that higher frequency words lead to faster RTs, but is in contrast with Libben and Titone (2008) and van Ginkel and Dijkstra (2020), who both reported that an increase in individual word frequency (verb and final noun frequency, respectively) led to slower RTs during idiom processing.

Remarkably, with respect to our third hypothesis about the semantic activation of individual words, we did not observe a facilitatory effect in the literal context for semantically related target words compared with unrelated target words. Although the 7 ms difference between the semantically related and unrelated target word was in the expected direction, it was not large enough to reach statistical significance. The absence of this effect is rather surprising because it has been reported in similar experimental paradigms with EEG or lexical decision (Federmeier, 2007; Federmeier et al., 2002; Federmeier & Kutas, 1999; Rommers et al., 2013). The lack of a facilitation effect in the literal context may be due to the nature of the task employed in our study, given that word naming does not explicitly demand the engagement of semantics.

However, another possibility is that the task was sensitive to semantics after all, but that in this naming task activation did not have enough time to spread from the correct and expected target word to semantically related words. Note that semantic priming effects are known to become stronger with increased prime durations (e.g., Holcomb et al., 2005; Lee et al., 1999) and longer stimulus onset asynchronies (SOAs) (e.g., Vorberg et al., 2004).

In fact, our word naming task *was* sensitive to semantics because we did find semantic effects of the idiom as a whole. In particular, in the idiomatic context, we observed an effect of idiom transparency (note that in the literal context the target words were not part of the idiom), even though only opaque idioms were included in our study. This facilitatory effect is in line with many studies on idiom processing (e.g., Gibbs, Nayak, & Cutting, 1989; Libben & Titone, 2008; van Ginkel & Dijkstra, 2020). When the individual words contribute to the figurative meaning, it is easier to process the idiom-final noun, as opposed to when the individual words do not contribute to the figurative meaning.

Given that our results were sensitive to semantic factors, at least in the idiomatic condition, we wanted to test whether the absence of effects in the literal context was due to timing aspects. Therefore, we conducted a second experiment in which we delayed the presentation of the target word relative to the rest of the sentence. This delay should give the target word’s activation more time to spread semantically to other words. As such, it should increase the chance of observing a facilitation effect for the semantically related target word in the literal context sentences.

## Experiment 2

### Methods

#### Participants

In total, 29 native speakers of Dutch participated in the experiment (22 females and 7 males). They were between 18 and 46 years old (*M* = 24.03, *SD* = 6.78) and had a normal or corrected-to-normal vision. They received compensation for participation in terms of a gift card or participant credits. Participants provided a written informed consent before the start of the experiment. This study was ethically assessed and approved by the EAC of the Faculty of Arts of Radboud University Nijmegen (number 3382).

#### Materials and design

The same materials and design as in Experiment 1 were used.

#### Procedure

Almost the same procedure as in Experiment 1 was used. The experiment consisted of two parts: a familiarisation phase and the main experiment. Experiment 2 differed from Experiment 1 with respect to the presentation of the target words in the main experiment. Similar to Experiment 1, sentences were presented visually in a word-by-word fashion presenting each word for 300 ms followed by a blank screen for 300 ms. However, the target word was not presented after a 300-ms blank screen, as in Experiment 1, but instead was delayed and displayed after a 500-ms blank screen.

#### Data analysis

The same procedure as in Experiment 1 was used to analyse the data.

### Results

Naming errors and trials with naming latencies shorter than 360 ms and longer than 1333 ms were removed from the data (7.0 %). Three participants were removed because of poor performance on the comprehension questions (<70% correct). Responses at 2.5 *SD*s from the mean were removed on the participant and item level (2.1%). The average naming latencies and *SD*s per Context and Condition are presented in Table 5.

**Table 5.** Mean naming latencies and *SD*s in Experiment 2.

Condition	Context	
	Literal	Idiomatic
COR	542 (113)	531 (105)
REL	568 (105)	565 (98)
UNREL	585 (113)	566 (101)

We analysed the naming latencies by means of a linear mixed-effects regression analysis with the logged naming latencies as the dependent variable. The final model consisted of the following fixed factors: (1) Trial Number (standardised), (2) Cloze Probability, (3) Sentence Plausibility, (4) Initial Sound (reference category: Fricatives), (5) Target Word Length, (6) Target Word Frequency (logged and mean centred), (7) Context (reference category: Literal), (8) Condition (reference category: REL), (9) Context × Condition, (10) Context × Target Word Frequency, (11) Condition × Target Word Frequency, and (12) Context × Condition × Target Word Frequency. As random effects, we included Participant (intercept and random slope of Trial Number) and Target Word (intercept only). The model is presented in Table 6.

**Table 6.** Regression model Experiment 2 with logged naming latencies as the dependent variable.

Fixed effects		Beta	*SE*	*t* value	
(Intercept)		6.3219	0.0314	201.093	***
Trial Number		−0.0046	0.0043	−1.069	
Cloze Probability		−0.0635	0.0150	−4.230	***
Sentence Plausibility		−0.0006	0.0024	−0.260	
Initial Sound (Vowels)		0.0586	0.0126	4.659	***
Initial Sound (Plosives)		0.0393	0.0072	5.480	***
Initial Sound (Nasals)		0.0101	0.0115	0.877	
Initial Sound (Approximants)		0.0082	0.0117	0.706	
Target Word Length		0.0075	0.0025	3.061	**
Target Word Frequency (TW freq)		−0.0031	0.0040	−0.775	
Context (Idiomatic)		−0.0062	0.0079	−0.790	
Condition (COR)		−0.0461	0.0115	−4.002	***
Condition (UNREL)		0.0289	0.0100	2.896	**
Context (Idiomatic) × Condition (COR)		−0.0171	0.0100	−1.710	.
Context (Idiomatic) × Condition (UNREL)		−0.0219	0.0103	−2.131	*
Context (Idiomatic) × TW freq		−0.0116	0.0043	−2.701	**
Condition (COR) × TW freq		−0.0081	0.0064	−1.277	
Condition (UNREL) × TW freq		0.0004	0.0058	0.069	
Context (Idiomatic) × Condition (COR) × TW freq		0.0302	0.0069	4.391	***
Context (Idiomatic) × Condition (UNREL) × TW freq		0.0060	0.0063	0.960	
Random effects		Variance	*SD*	Corr.	
Target word	Intercept	0.0005	0.0215		
Participant	Intercept	0.0141	0.1189		
	Trial number	0.0004	0.0192	−0.08	
Residual		0.0166	0.1286		

* *p* < .05. **p < .01. ***p < .001.

**Figure 1.**
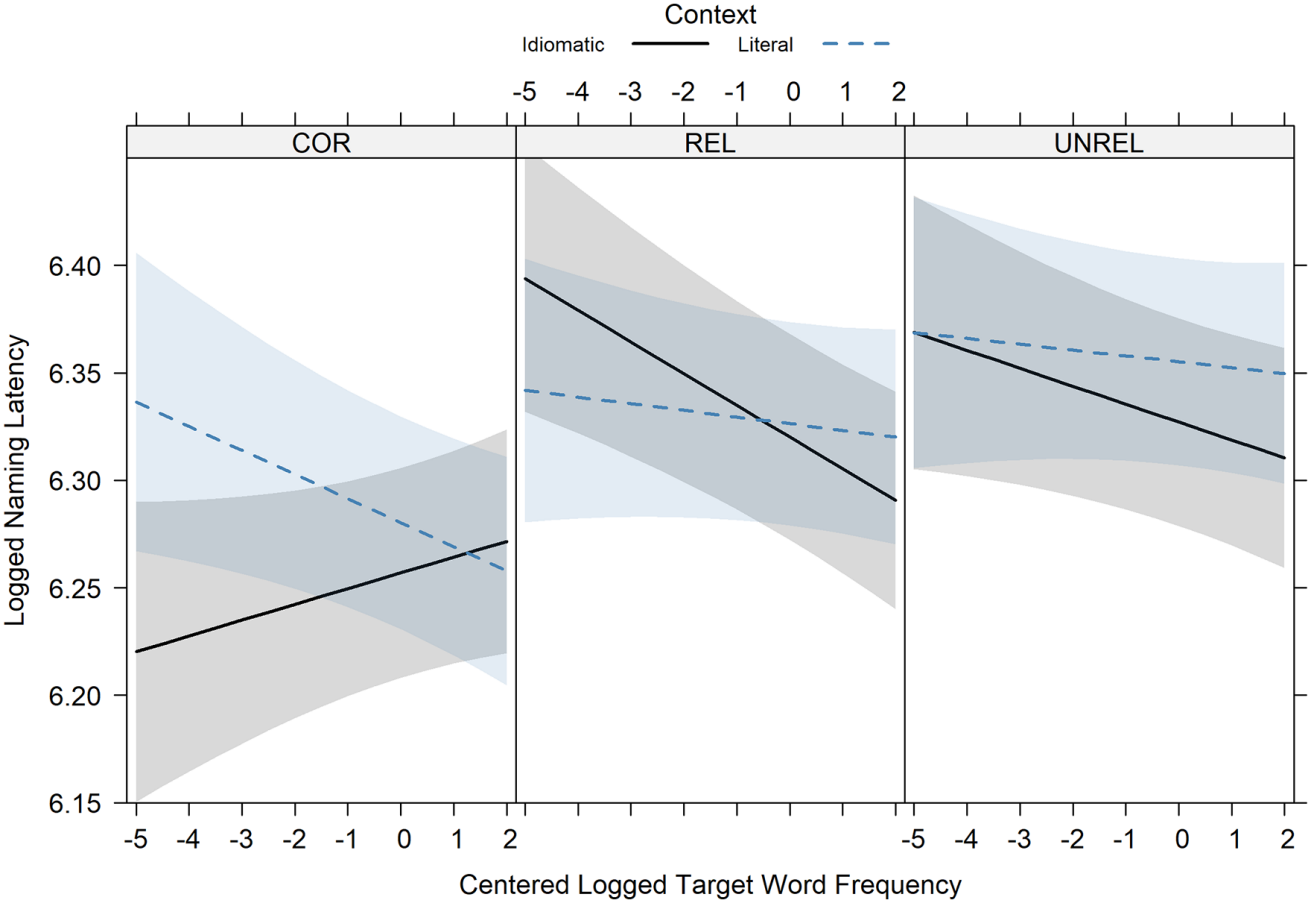
The effect of target word frequency by context and condition. The error bands are based on *SE*s.

This analysis revealed an interesting significant three-way interaction with Target Word Frequency, Context, and Condition. More specifically, the effect of Target Word Frequency on naming latencies was different for the correct target word as opposed to the semantically related target word in the idiomatic context, but not in the literal context (β = 0.030, *SE* = 0.007, *p* < .001). The interaction effect is visualised in Figure 1.

Moreover, the Cloze Probability of the correct target word also significantly affected naming latencies (β = −0.064, *SE* = 0.015, *p* < .001). Sentences in which the correct target word was more predictable elicited faster naming latencies than sentences in which the correct target word was less predictable, irrespective of the Context and Condition.

Adding idiom properties (in interaction with Context) did not significantly affect the naming latencies, as this did not lead to an improved model fit.

#### Separate analyses

To obtain a better insight into the three-way interaction effect, we analysed the idiomatic and literal contexts separately. For both sub-analyses, a linear mixed-effects regression analysis was carried out including the same random and fixed factors as in the regression model based on the complete dataset except Context.

The following fixed factors were included: (1) Trial Number (standardised), (2) Cloze Probability, (3) Sentence Plausibility, (4) Initial Sound (reference category: Fricatives, (5) Target Word Length, (6) Target Word Frequency (logged and mean centred), (7) Condition (reference category: REL), and (8) Target Word Frequency × Condition. As random effects we included Participant (intercept and random slope of Trial Number) and Target Word (intercept only). The regression models based on the Literal and Idiomatic Context Sentences are presented in Tables 7 and 8, respectively.

**Table 7.** Regression model Experiment 2 for the literal context sentences only with logged naming latencies as the dependent variable.

Fixed effects		Beta	*SE*	*t* value	
(Intercept)		6.2723	0.0358	175.178	***
Trial Number		−0.0062	0.0048	−1.288	
Cloze Probability		−0.0264	0.0209	−1.261	
Sentence Plausibility		0.0027	0.0028	0.933	
Initial Sound (Vowels)		0.0631	0.0143	4.401	***
Initial Sound (Plosives)		0.0474	0.0082	5.806	***
Initial Sound (Nasals)		−0.0008	0.0132	−0.057	
Initial Sound (Approximants)		0.0142	0.0133	1.064	
Target Word Length		0.0084	0.0028	3.004	**
Target Word Frequency		−0.0015	0.0039	−0.396	
Condition (COR)		−0.0548	0.0120	−4.553	***
Condition (UNREL)		0.0348	0.0100	3.469	***
Condition (COR) × Target Word Frequency		−0.0100	0.0062	−1.618	
Condition (UNREL) × Target Word Frequency		−0.0018	0.0056	−0.318	
Random effects		Variance	*SD*	Corr.	
Target word	Intercept	0.0004	0.0186		
Participant	Intercept	0.0162	0.1274		
	Trial number	0.0004	0.0196	0.09	
Residual		0.0169	0.1298		

* *p* < .05. **p < .01. ***p < .001.

**Table 8.** Regression model Experiment 2 for the idiomatic context sentences only with logged naming latencies as the dependent variable.

Fixed effects		Beta	*SE*	*t* value	
(Intercept)		6.3426	0.0374	169.542	***
Trial Number		−0.0030	0.0047	−0.639	
Cloze Probability		−0.0811	0.0252	−3.213	
Sentence Plausibility		−0.0018	0.0051	−0.349	
Initial Sound (Vowels)		0.0559	0.0162	3.461	***
Initial Sound (Plosives)		0.0325	0.0092	3.540	***
Initial Sound (Nasals)		0.0152	0.0147	1.032	
Initial Sound (Approximants)		0.0032	0.0148	0.219	
Target Word Length		0.0061	0.0032	1.924	.
Target Word Frequency		−0.0159	0.0044	−3.642	***
Condition (COR)		−0.0590	0.0225	−2.627	**
Condition (UNREL)		0.0053	0.0097	0.544	
Condition (COR) × Target Word Frequency		0.0218	0.0067	3.241	**
Condition (UNREL) × Target Word Frequency		0.0070	0.0061	1.140	
Random effects		Variance	*SD*	Corr.	
Target word	Intercept	0.0006	0.0248		
Participant	Intercept	0.0121	0.1099		
	Trial number	0.0004	0.0192	−0.27	
Residual		0.0160	0.1266		

* *p* < .05. **p < .01. ***p < .001.

In the analysis based on the Literal context sentences only, we found no significant interaction effect between Condition and Target Word Frequency (β = −0.010, *SE* = 0.006, *p* > .05 and β = −0.002, *SE* = 0.006, *p* > .05). However, a facilitatory effect of Target Word Frequency was observed for correct target words (relevelled version of the model: β = −0.018, *SE* = 0.005, *p* < .05). Crucially, the analysis revealed significant differences between COR, REL, and UNREL. Participants were significantly slower in response to semantically related target words than to their correct counterparts (β = −0.055, *SE* = 0.012, *p* < .001), but faster than in response to the semantically unrelated target words (β = 0.035, *SE* = 0.010, *p* < .001). In addition, significant covariates were Target Word Length and Initial Sound. The longer the target words, the longer the naming latencies (β = 0.008, *SE* = 0.003, *p* < .01), and target words starting with a fricative were named faster than target words starting with a vowel (β = 0.063, *SE* = 0.014, *p* < .001) and a plosive (β = 0.047, *SE* = 0.008, *p* < .001). No significant effects were found for Cloze Probability and Sentence Plausibility.

The regression model based on the Idiomatic context sentences only (see Table 8) revealed a significant interaction effect between Condition and Target word frequency. The effect of Target Word Frequency was significantly different for correct words as opposed to semantically related words (β = 0.022, *SE* = 0.007, *p* < .01) and semantically unrelated target words (relevelled version of the model: β = 0.015, *SE* = 0.007, *p* < .05). The effect of Target Word Frequency on naming latencies was similar for semantically related and unrelated target words (β = 0.007, *SE* = 0.006, *p* > .05). Interestingly, naming latencies for correct target words were significantly faster than for semantically related target words (β = −0.059, *SE* = 0.022, *p* < .01), whereas naming latencies for semantically related and unrelated target words did not differ (β = 0.005, *SE* = 0.010, *p* > .05). A relevelled version of the model showed that naming latencies for correct target words were also significantly faster than naming latencies for semantically unrelated target words (β = −0.064, *SE* = 0.024, *p* < .01). In addition, we found a facilitatory effect of Cloze Probability, indicating that for sentences in which the correct target word was more predictable, the naming latencies were faster than for sentences in which the correct target word was less predictable (β = −0.081, *SE* = 0.025, *p* < .01) Moreover, naming latencies in response to target words starting with a fricative were different from naming latencies in response to target words starting with a vowel (β = 0.046, *SE* = 0.016, *p* < .01) or a plosive (β = 0.030, *SE* = 0.010, *p* < .01). Sentence plausibility did not significantly affect naming latencies.

### Discussion

In line with Experiment 1, participants responded faster to the correct target word than to unrelated target words in both the literal and the idiomatic sentence contexts. This finding suggests that participants used the sentence context for faster integration of its final word. With respect to the idiomatic context, this is evidence that the idioms were activated as a whole and were recognised at the form level, as stated in our first prediction.

In addition, we observed activation of the orthographic form of the target word, as witnessed by an effect of individual orthographic word frequency. More specifically, higher target word frequencies were associated with longer naming latencies of the idiom-final noun in the idiomatic context, while in the literal context higher target word frequencies did not lead to shorter naming latencies for the correct target word. This is in support of our second prediction and suggests that a higher individual word frequency may hinder idiom processing. This inhibitory effect on idiom processing is in line with the verb frequency effect reported by Libben and Titone (2008) and with van Ginkel and Dijkstra (2020), who observed a comparable effect of the idiom-final noun frequency. Thus, although the idiom-final noun was apparently not activated strongly enough to spread semantic activation to related words, participants still accessed form aspects of this word related to its literal use even in a strongly idiomatically biasing context containing opaque idioms.

Note that in Experiment 2 we observed faster naming latencies in the literal context for semantically related words than for unrelated words. Similar results were obtained in earlier studies using this paradigm with lexical decision and EEG (Federmeier, 2007; Federmeier et al., 2002; Federmeier & Kutas, 1999; Rommers et al., 2013). Apparently, a delayed target word presentation of 200 ms was enough to increase the activation of the correct target word to such an extent that it was able to spread semantic activation to related words.

For the idiomatic context, we found no facilitation of semantically related target words compared with unrelated words. This, in combination with the presence of the effect in the literal context, supports our prediction that the literal word meanings are not activated to a large extent during the processing of opaque Dutch idioms. In other words, the facilitatory effect due to semantic relatedness in the idiomatic context may have been reduced because the literal meaning of the idiom-final noun was suppressed.

## General discussion

In two experiments, we studied how opaque idioms and their individual words are activated and processed both orthographically and semantically. Target words were presented at the end of idiomatic and literal sentence contexts, following the presentation paradigm by Rommers et al. (2013). However, in contrast to this earlier study, participants named target words at the end of literally and idiomatically biasing sentences, rather than making lexical decisions on them. A word naming task relies more on orthographic and phonological word form than lexical decision, which may be more sensitive to both orthography and semantics. Therefore, effects of individual word meanings were expected to be reduced in word naming, while information related to the word form, such as frequency of word usage, might be more prominent.

In two experiments, we tested three hypotheses: (1) representations of idioms as a whole are activated and recognised during processing; (2) individual words at the end of idiomatically biasing sentences are activated at least orthographically and phonologically because they must be identified to verify that the idiom is actually present; and (3) because the meanings of individual target words are inconsistent with the idiom’s meaning, they are suppressed at the end of idioms but not in literally biasing sentences.

In our first experiment, participants responded faster to correct target words at the end of both idiomatic and literal contexts compared with unrelated target words. Even when the target word presentation was delayed by 200 ms, as in Experiment 2, participants still responded faster to these words. This finding supports our first hypothesis that idioms have separate representations that are activated and recognised during sentence processing.

Our evidence further indicates that in idiomatically biasing sentences, individual target words were activated in parallel with these idiom representations. In particular, in line with our second hypothesis, in an idiomatic sentence context, the idiom’s final noun target still appeared to be activated orthographically. Evidence for this assertion was the presence of a significant effect of orthographic target word frequency. For instance, in Experiment 1, a higher item frequency was associated with a faster response. Interestingly, the idiom-final noun must also have been activated at the form level in Experiment 2, but here higher idiom-final noun frequencies led to slower naming latencies. Generally, higher word frequencies are associated with faster processing times (see Brysbaert et al., 2018, for a review). Nevertheless, there is a limited number of studies on the role of single word frequency in idiom processing (Libben & Titone, 2008; van Ginkel & Dijkstra, 2020) that report similar results to ours. The inhibitory effect can be seen as an indication of competition between the idiom-final noun and the idiom as a whole. The change from a facilitatory to an inhibitory effect of word frequency going from Experiment 1 to Experiment 2 could be explained by assuming changes in the relative activation of idiom and individual word representations over time. Early on, a high-frequency item is more quickly activated than a low-frequency item, which could lead to a faster co-activation of an idiomatic representation. Thus, a facilitation effect might be expected. Later in time, however, a high-frequency item would be more competitive with the idiomatic representation, resulting in an inhibitory effect.

Related to this point, our third hypothesis was that the meaning of an individual target word would be suppressed at the end of idioms because the word’s meaning was inconsistent with that of the idiom at hand. In contrast, in literal sentences, this meaning would remain active. Indeed, in Experiment 1 we found that RTs for the final noun in an idiom were non-significantly different for targets related and unrelated to the correct item (COR < REL = UNREL). However, the same non-significant difference was obtained for target words in literal sentences. Thus, we did not find the graded pattern of results that was reported by Rommers et al. (2013) in a literal context. In preparation for Experiment 2, we considered two explanations for this finding. First, responses in the REL condition might have been already as fast as they could be (floor effect) because word naming is generally faster than lexical decision as used by Rommers et al. (2013). Alternatively, the temporal settings for naming responses may have been affected by the presence of a mixed stimulus list. Whatever the correct explanation was, the observed absence of facilitation was not a problem of insensitivity to semantics. Note that while no semantic effects were observed of the target words in Experiment 1, we did find effects related to the semantics of the idiom as a whole. Idiom transparency turned out to affect idiom processing. In line with previous studies, more transparent idioms led to faster RTs than less transparent idioms (Gibbs, Nayak, & Cutting, 1989; Libben & Titone, 2008; van Ginkel & Dijkstra, 2020).

Under the assumption that there was not enough time to spread activation due to insufficient activation and/or fast responses, an effect should emerge when the target word was presented at a delay of 200 ms because semantic priming is found to become stronger with increased prime durations (e.g., Holcomb et al., 2005; Lee et al., 1999) and longer SOAs (e.g., Vorberg et al., 2004). This was the main manipulation of Experiment 2.

Delaying the presentation of the target word by 200 ms in Experiment 2 did indeed lead to a graded pattern for target word condition (COR < REL < UNREL) in the literal context that had been absent in Experiment 1. This pattern points at pre-activation of the correct word and subsequent spreading activation to semantically related words. Importantly, naming latencies for the semantically related and unrelated words in the idiomatic context did not differ. These findings together support the view that here the idiom-final nouns were not activated at the semantic level. In the context of an idiom, facilitatory effects of the target word’s spreading activation were apparently reduced or cancelled out by suppression of the individual word meanings.

The manipulation of the moment that the target word was presented (immediately in Experiment 1 and after 200 ms in Experiment 2) allows us to formulate a time-course of activation at the orthographic and semantic levels. In line with van Ginkel and Dijkstra (2020), we argue that in the word-by-word presentation of an idiomatic phrase, the figurative meaning representation builds up over time, as more and more information becomes available. The representation is completed once the last word is presented. This completion process requires the word form, but not the word meaning.

Our findings confirm this line of reasoning. In a strongly idiomatically biasing context containing opaque idiomatic expressions, the word meaning of the idiom-final noun is suppressed because it does not contribute to the figurative meaning representation. However, the word form needs to be checked, which results in activation of the word form as confirmed by a word frequency effect. More specifically, the idiom-final noun is in competition with the idiom as a whole at the orthographic level. Higher idiom-final noun frequencies lead to more difficulties in integrating the idiom-final noun into the idiomatic context.

This explanation is, of course, strongly related to the methodology we adopted. In our study, sentences were presented word by word, and the target word had to be integrated into the sentence context, as it was the final word of the sentence. Right before presenting this final word, participants were not certain the sentence would be idiomatic, especially because the idiom-final noun was actually presented only in one third of the cases. In the other cases, this word was replaced by a semantically related or unrelated word. As a consequence, participants did need the idiom-final word to complete the idiom representation, which might explain the competition between the idiom-final noun and the idiom as a whole at the orthographic level.

Other studies, applying different techniques, may find different results and draw different conclusions about the role of the individual words in idiom processing. The crucial difference between our study and studies that used the cross-model priming paradigm, for example, is related to the moment in time responses to target words are measured. In such cross-modal priming experiments, RTs are measured on (visually presented) target words that were not part of the prime sentence (Cacciari & Tabossi, 1988; Titone & Libben, 2014; van Ginkel & Dijkstra, 2020). In these studies, the idiom is already presented in full as part of the prime sentence. Hence, the idiom representation is retrieved when the target word is processed. These task differences complicate a comparison of studies (as mentioned also by van Ginkel & Dijkstra, 2020) because idiom processing and the role of the individual words have been investigated at different points in time. This suggests that more research is needed that systematically disentangles task effects on idiom processing.

In any case, our time-course analysis proposed above is in line with some current models for idiom and literal sentence processing, but less so with others. In particular, our results are in line with so-called hybrid models of idiom processing. According to hybrid models, idiomatic expressions are stored in the mental lexicon as a whole (e.g., Sprenger et al., 2006). In our study, the idiom as a whole is activated and recognised during sentence processing, while the idiom-final noun is suppressed semantically. However, even when opaque idiomatic expressions are embedded in a strongly idiomatically biasing context, traces of individual word form activation are found in terms of orthographic word frequency effects. This finding supports the view that figurative and literal processing run in parallel.

In fact, all our major findings are in line with the hybrid model by Sprenger et al. (2006) when it is applied to idiom comprehension. According to this model, the idiom has a separate representation (*superlemma*) that is connected to its corresponding idiomatic meaning, on the one hand, and to simple word lemmas, on the other hand. The superlemmas can be accessed by activating these simple lemmas. The superlemma, in turn, activates the corresponding idiom meaning representation. In the context of our study, the simple word lemmas have to be activated because the incoming target word needs to be checked to determine whether it is part of an idiom. However, the corresponding concepts can be ignored because of the opacity of our idiomatic expressions, that is, individual word meanings did not contribute to the figurative meaning. This effect is probably strengthened by the highly idiomatically biasing context in which the idioms were presented. Therefore, the individual words were not activated semantically, while evidence for orthographic activation was observed in terms of word frequency effects.

We conclude that our results argue convincingly against purely compositional and non-compositional models of idiom processing. On the one hand, according to compositional models, the individual word meanings are accessed and combined to retrieve the figurative meaning (Cacciari & Glucksberg, 1991; Gibbs, Nayak, Bolton, & Keppel, 1989; Nunberg, 1979). In this study, however, the individual words were apparently not accessed at the semantic level because facilitation of the semantically related word was absent in Experiment 2.

On the other hand, non-compositional models argue that idioms are stored as a whole in the mental lexicon and that individual word meanings are not activated during processing (Bobrow & Bell, 1973; Gibbs, 1980; Swinney & Cutler, 1979). Our study shows that this is not the case either. Although individual words were suppressed at the semantic level, traces of activation at the word form level were found, as reflected by word frequency effects.

## Conclusion

Using a word naming task, we investigated to what extent individual words at the end of sentences are activated semantically and orthographically during the processing of opaque Dutch idiomatic expressions. In an idiomatic sentence context, where word semantics do not contribute to the figurative meaning, correct target words were responded to faster than targets related in meaning or unrelated. This suggests that individual word meanings were suppressed or not activated substantially at the time of responding. However, the idiom-final noun was active at the orthographic-phonological level, as indicated by word frequency effects. Note that the form representation is required to verify that an idiom was actually being presented and to comply with the demands of the word naming task. Time-course aspects of activation were investigated in a second experiment. When the presentation of the sentence-final target noun was delayed by 200 ms (from 300 to 500 ms), a semantic facilitation effect appeared for the correct and expected word (COR) that was indicative of spreading activation. Together these results support a hybrid model of idiom processing in which individual words and the idiom as a whole interact at both orthographic and semantic levels of representation.

## Supplemental Material

sj-docx-1-qjp-10.1177_17470218211047995 – Supplemental material for Individual word activation and word frequency effects during the processing of opaque idiomatic expressions Click here for additional data file. Supplemental material, sj-docx-1-qjp-10.1177_17470218211047995 for Individual word activation and word frequency effects during the processing of opaque idiomatic expressions by Ferdy Hubers, Catia Cucchiarini, Helmer Strik and Ton Dijkstra in Quarterly Journal of Experimental Psychology
