# Supplementary material for: Individual word activation and word frequency effects during the processing of opaque idiomatic expressions
Source: Q J Exp Psychol (Hove). 2021 Oct 4;75(6):1004–20. doi: 10.1177/17470218211047995 (PMC9016674; doi:10.1177/17470218211047995)
Supplement: sj-docx-1-qjp-10.1177_17470218211047995 – Supplemental material for Individual word activation and word frequency effects during the processing of opaque idiomatic expressions [file sj-docx-1-qjp-10.1177_17470218211047995.docx]

**Supplementary Materials**

Individual Word Activation and Word Frequency Effects during the Processing of Opaque Idiomatic Expressions

| *Table S1*  *An Overview of the Dutch Idiomatic Expressions and the Target Words used in Experiments 1 and 2.* | | | | | |
| --- | --- | --- | --- | --- | --- |
| **#** | **Idiomatic expression** | **Meaning** | **COR** | **REL** | **UNREL** |
| 1 | aan de grond zitten | in slechte omstandigheden verkeren | grond | lucht | droom |
| 2 | bij iemand een wit voetje halen | bij iemand in de gunst proberen te komen | voetje | teentje | bosje |
| 3 | boter bij de vis | contant betalen | vis | zee | grap |
| 4 | de kat op het spek binden | iemand in verleiding brengen | spek | vet | gaas |
| 5 | een klein hartje hebben | gauw bang zijn | hartje | kusje | hoekje |
| 6 | een slag om de arm houden | iets onder voorbehoud afspreken | arm | pols | rust |
| 7 | een vinger in de pap hebben | invloed hebben op iets | pap | melk | krant |
| 8 | een wassen neus | niet van belang | neus | oog | trein |
| 9 | hek van de dam | geen belemmeringen meer hebben | dam | bever | dolk |
| 10 | het op zijn heupen krijgen | plotseling fanatiek bezig gaan | heupen | billen | bedden |
| 11 | hoog van de toren blazen | opscheppen | toren | klok | leden |
| 12 | iemand iets in de maag splitsen | iemand iets dwingen te doen | maag | buik | huur |
| 13 | iemand iets op de mouw spelden | iemand iets wijsmaken | mouw | trui | slee |
| 14 | iemand in de kaart spelen | iemand onbedoeld helpen | kaart | brief | helft |
| 15 | iemand om zeep helpen | iemand vermoorden | zeep | sop | munt |
| 16 | iets onder de knie hebben | iets goed kunnen | knie | schijf | villa |
| 17 | iets soldaat maken | zich arrogant gedragen | soldaat | majoor | getuige |
| 18 | iets uit de doeken doen | iets uitleggen | doeken | lakens | herten |
| 19 | koek en ei zijn | goede vrienden zijn | ei | ham | zaal |
| 20 | lange tenen hebben | snel beledigd zijn | tenen | nagels | apen |
| 21 | met zijn neus in de boter vallen | in een gunstige situatie terechtkomen | boter | pan | klem |
| 22 | naast zijn schoenen lopen | zich arrogant gedragen | schoenen | benen | partners |
| 23 | op de fles gaan | failliet gaan | fles | drank | tuin |
| 24 | op de tocht staan | in een bedreigde positie komen | tocht | kou | lente |
| 25 | op een laag pitje staan | minder aandacht krijgen | pitje | vlammetje | kiertje |
| 26 | op zijn strepen staan | zijn eigen mening aanhouden | strepen | lijnen | messen |
| 27 | tegen de lamp lopen | betrapt worden | lamp | warmte | helm |
| 28 | tegen het plafond zitten | niet meer kunnen bereiken | plafond | dak | fornuis |
| 29 | veel voeten in de aarde hebben | veel moeite kosten | aarde | hemel | onzin |
| 30 | voor spek en bonen | zonder mee te tellen | bonen | erwten | beren |
